# Scanning transmission helium ion microscopy on carbon nanomembranes

**DOI:** 10.3762/bjnano.12.18

**Published:** 2021-02-26

**Authors:** Daniel Emmrich, Annalena Wolff, Nikolaus Meyerbröker, Jörg K N Lindner, André Beyer, Armin Gölzhäuser

**Affiliations:** 1 Physics of Supramolecular Systems and Surfaces, Bielefeld University, 33615 Bielefeld, Germany; 2 Central Analytical Research Facility, Institute for Future Environments, Queensland University of Technology, 2 George St, Brisbane 4000, QLD, Australia; 3 CNM Technologies, Bielefeld, Germany; 4 Department of Physics, Paderborn University, Paderborn, Germany

**Keywords:** carbon nanomembranes, dark field, helium ion microscopy (HIM), scanning transmission ion microscopy (STIM), SRIM simulations

## Abstract

A dark-field scanning transmission ion microscopy detector was designed for the helium ion microscope. The detection principle is based on a secondary electron conversion holder with an exchangeable aperture strip allowing its acceptance angle to be tuned from 3 to 98 mrad. The contrast mechanism and performance were investigated using freestanding nanometer-thin carbon membranes. The results demonstrate that the detector can be optimized either for most efficient signal collection or for maximum image contrast. The designed setup allows for the imaging of thin low-density materials that otherwise provide little signal or contrast and for a clear end-point detection in the fabrication of nanopores. In addition, the detector is able to determine the thickness of membranes with sub-nanometer precision by quantitatively evaluating the image signal and comparing the results with Monte Carlo simulations. The thickness determined by the dark-field transmission detector is compared to X-ray photoelectron spectroscopy and energy-filtered transmission electron microscopy measurements.

## Introduction

Throughout the past decade, the helium ion microscope (HIM) has emerged as a versatile instrument that is used to drive research across multiple disciplines. While it can be operated like a scanning electron microscope (SEM) for imaging applications, it offers a higher resolution at a larger depth of field than SEMs [1]. A major advantage of the technology besides its surface-sensitive imaging capability is the ability to record charge-compensated images on insulating samples such as biological specimen or polymers without requiring a conductive coating layer [2–4].

In addition, the ability to record images with high signal-to-noise ratio while using low beam currents (the HIM creates up to five times more secondary electrons (SE) than an SEM [5]) is advantageous when working with beam-sensitive samples. An overview of the imaging as well as recently added analytical capabilities using secondary ion mass spectroscopy can be found in a recent review [6].

Beyond imaging, the HIM has been established as a key nanofabrication tool for milling [7–9], defect engineering [10–11], and resist-based lithography [12–13], overcoming the resolution limitations of other FIB techniques [14–15].

Both bulk samples as well as thin membranes have been structured using the HIM. On membranes, the sputter yield is significantly increased because sputtering occurs not only in backward but also in forward direction [16–17]. To observe and control the milling process, the ion transmission signal is preferred over the SE signal because it is related to the membrane thickness. The detection of the transmission signal can be achieved in different ways. In a SE conversion plate holder, the sample is placed above a polished metal plate that is turned towards the SE detector of the microscope. Transmitted ions release SE from the plate while apertures on the polished metal plate filter bright-field and dark-field signal, similar to scanning transmission electron microscopy (STEM). In this way, it is possible to check qualitatively the milling progress on membranes or even to determine quantitatively the thickness of a sample. Notte et al. used thickness fringes on MgO crystals for thickness determination [18], Hall measured the thickness of a silicon nitride membrane down to 5 nm using the bright-field signal [19]. A different detection method is the use of a microchannel plate (MCP). Woehl et al. were able to resolve the core–shell structure of silica-coated gold nanoparticles with an annular detector in the dark field [20]. Kavanagh et al. used a silicon diode array as a pixelated sensor for transmission imaging to observe ion beam scattering with a static beam and as an end-point detection for pore milling into graphite sheets [21].

This work presents the design and capabilities of a dark-field scanning transmission ion microscopy (STIM) holder. The holder design is based on the concept of a SE conversion holder. The holder can easily be implemented into any existing HIM. It is mounted on the sample stage without modifications to the microscope.

Carbon nanomembranes (CNMs) serve as test samples. These membranes are made from aromatic molecules, typically from self-assembled monolayers. Upon electron irradiation, cross-linking of the molecules is induced. A mechanically stable membrane is formed, which can be transferred onto any other substrate, such as TEM grids [22–23]. Since CNMs originate from a molecular thin film, they can be as thin as 1 nm. The conductivity of such membranes is low, which poses a challenge to SE imaging with charged-particle microscopes such as SEM or HIM [24].

This work demonstrates that the holder is easily optimized for such difficult samples. It is possible to image a sample area and vary the acceptance angles from 3 to 98 mrad under constant microscope conditions. Thus, it is possible to study the behavior of the STIM signal at different acceptance angles.

## Results and Discussion

### STIM detector design

**Figure 1:**
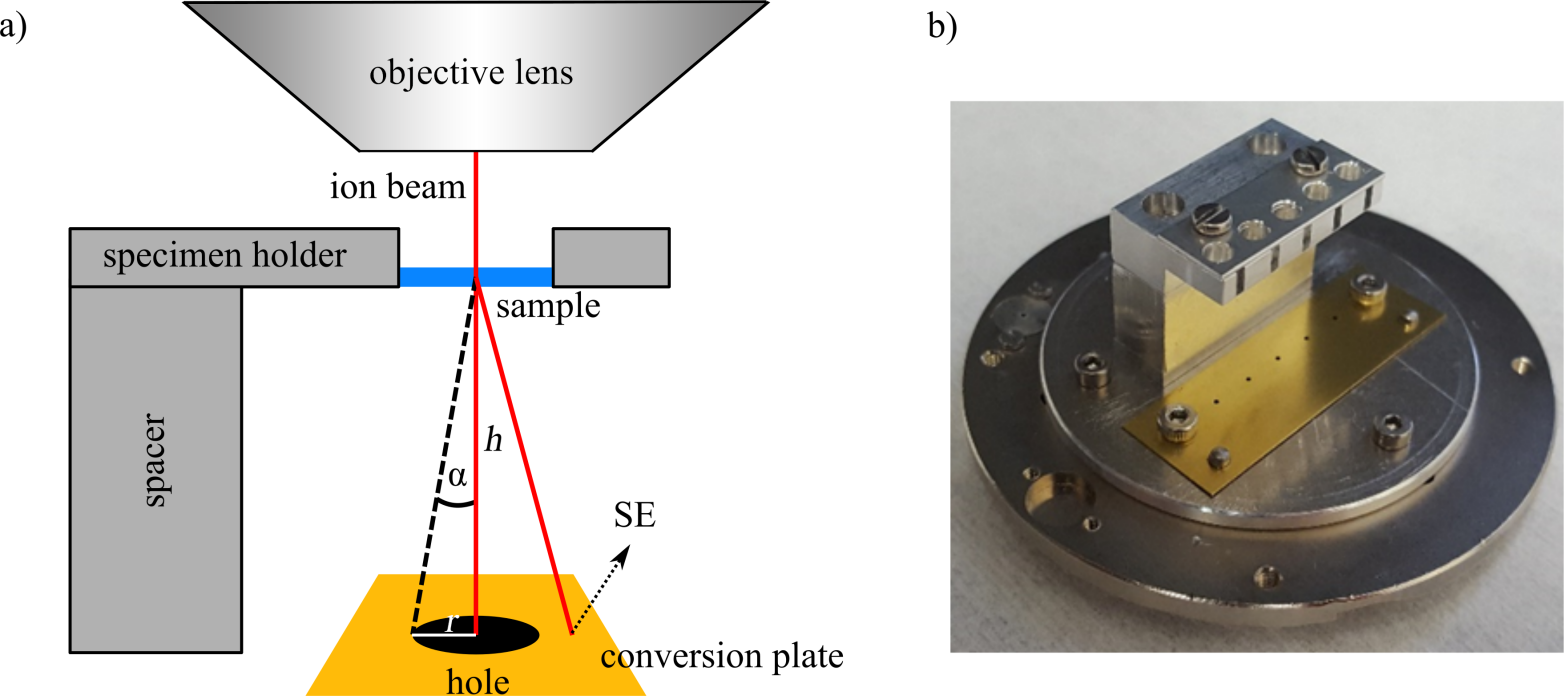
(a) Operating principle of the STIM dark-field detector. Thin foils or membranes are placed in the top part of the detector, which is mounted above the conversion plate at a specific height *h*. The height can be adjusted by an exchangeable spacer block. The metal conversion plate has differently sized holes directly underneath the mounted sample. Ion trajectories are altered during the ion–solid interaction and exit the thin foils or membranes at the bottom at different angles. The acceptance angle α depends on the height *h* and the radius of the hole *r*. Transmitted ions either enter the hole or they hit the metal conversion plate, which produces SE that can be detected with the ETD. (b) Photo of the detector for the HIM showing slots for five samples.

The designed dark-field STIM detector (Figure 1a) is based, in principle, on a SE conversion holder. It is mounted as a specimen holder onto the microscope stage at which the beam is scanned over the sample. Multiple samples can be mounted at a distance above a brass plate. On this conversion plate, ions release SE, which are detected by the Everhart–Thornley detector (ETD). Ions that are transmitted through a thin specimen (e.g., a membrane), undergo elastic and inelastic scattering processes, which lead to deflection of the ion trajectories. Depending on the resulting scattering angle, the transmitted ions hit different points on the conversion plate below the sample. This plate has holes centered underneath each specimen position. The holes act as Faraday cups. Ions that are deflected by a low angle will pass the hole and do not create a signal on the conversion plate. Ions that are deflected by large angles will hit the conversion plate and create a signal that can be detected with the ETD. Therefore, the brightness of a pixel in the final image is determined by the degree of scattering. Additionally, the brightness is determined by the acceptance angle α of the STIM holder. This angle is defined by the size of the hole on the conversion plate. It is the minimum deflection angle for ions to create a transmission signal and can be calculated using

[1] tanα=r/h,

with α as the acceptance angle, *h* as the operational height of the holder (i.e., the distance between a thin membrane and the conversion plate) and *r* as the radius of the hole (Figure 1a). An increasing acceptance angle results in an overall darker image as more and more scattered ions are excluded from the STIM signal.

The dark-field STIM holder (Figure 1b) allows one to load up to five samples at one time. Each sample is mounted 15.3 mm above the conversion plate. Different conversion plates with hole diameters from 100 μm to 3 mm lead to acceptance angles between 3 and 98 mrad. Two centering pins ensure that all aperture strips are always mounted at the identical position on the holder. The total size of the holder is limited by the dimensions of the load lock valve as well as by the gap between holder in the specimen chamber and objective lens.

The required hole sizes depend on the deflection angles, which are determined by beam energy, sample thickness, and sample material. The angles can be predicted using Monte Carlo simulations, which yield information about the relevant range of hole diameters by employing Equation 1.

For quantitative analysis of the STIM signal, a proper alignment of the sample is crucial. A beam shift needs to be avoided to ensure a perpendicular beam on the sample and the imaging area must be centered above the hole in the conversion plate.

In order to measure the pure STIM signal, SE emitted directly from the sample need to be blocked. Ideally, the resulting signal consists exclusively of SE released by transmitted ions. Three measures were taken to filter out direct SE. Firstly, CNMs have a low conductivity, which leads to charging and reduces the SE emission from the sample surface [25]. In addition, the holder is set to a positive voltage of 180 V that pulls the SE away from the detector. Furthermore, the samples are placed in 2.5 mm deep boreholes for signal shadowing, while the holder is in close proximity to the objective lens.

**Figure 2:**
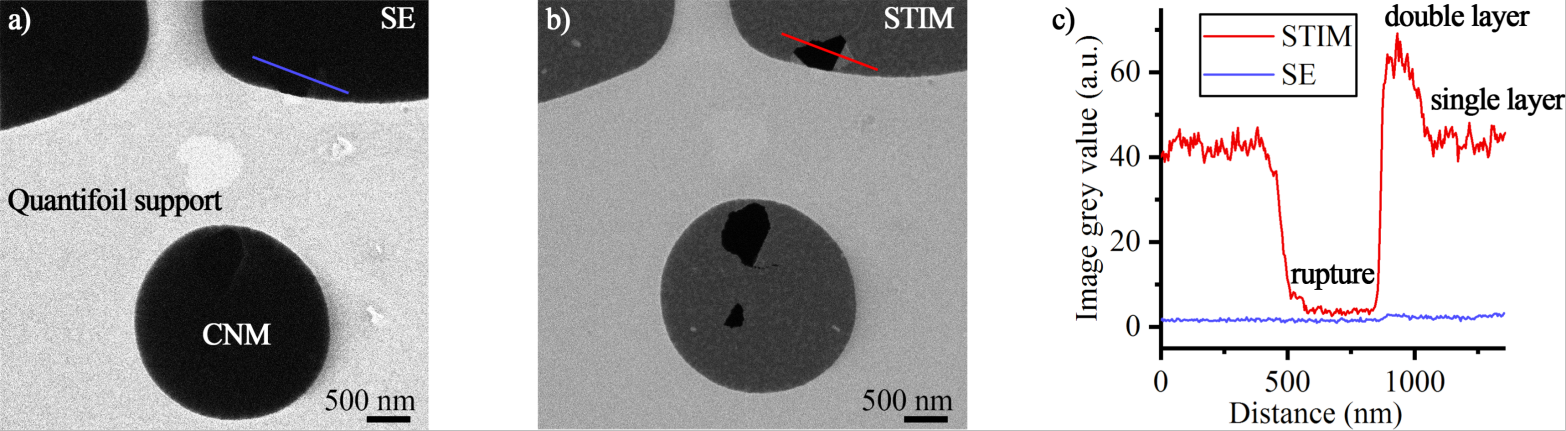
Comparison of HIM detection schemes. (a) SE and (b) dark-field STIM signal at an acceptance angle of 13 mrad show the strength of STIM on the CNM. Both images were recorded with the same ETD settings, but contrast was adjusted individually for publication. Line profiles from each raw signal (c) prove that in STIM configuration, the SE contribution originating directly from the sample is negligible. In the STIM analysis of CNM, the signal difference between rupture and double layer is one grey level in SE, compared to 57 grey levels in STIM, while there is no difference between single and double layer in SE configuration.

To visualize the SE contribution that is overlaying the STIM signal in later experiments, a CNM with a thickness of about 2 nm was imaged (Figure 2). This amorphous and insulating membrane is placed on a conductive Quantifoil TEM support grid. The sample was first imaged in a configuration that excludes all transmitted ions from the detection chain and leads to a SE signal only originating from the sample (Figure 2a). For this configuration, the aperture strip is removed, exposing a large hole in the base plate, and the spacer block (see Figure 1) is removed to mount the specimen holder directly over the hole. In Figure 2b the same sample area was imaged with the holder in STIM configuration as depicted in Figure 1, that is, spacer block and aperture strip were installed. In both configurations a stage bias of 180 V was applied. While the free-standing CNM shows nearly no signal in the SE configuration, the STIM signal reveals details about ruptures and folds in the membrane. The conducting Quantifoil support is visible in both configurations, with an average grey level of 23 in SE and 117 in STIM, suggesting a significant amount of direct SE in STIM configuration in this region. STIM analysis will only be conducted on areas of freestanding CNM. Intensity profiles across a feature (a rupture and a fold) show that the portion of direct SE from the CNM is negligible for the STIM analysis (Figure 2c).

### Monte Carlo simulations and signal/contrast modelling

**Figure 3:**
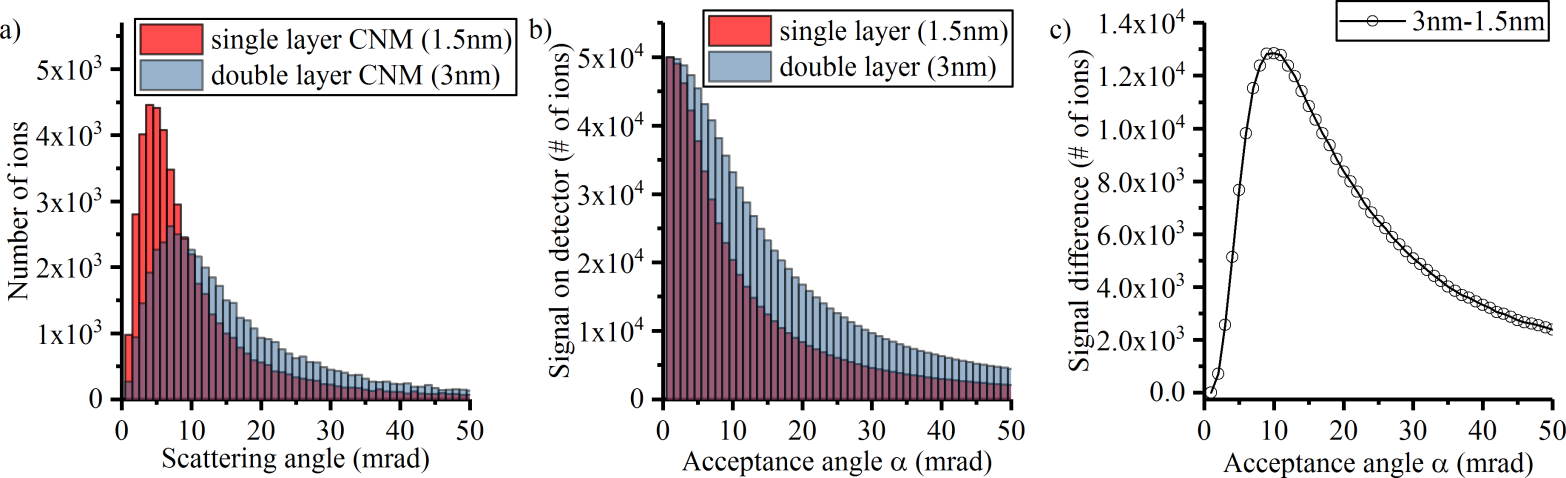
(a) SRIM scattering distribution of 30 keV He ions for single- and double-layer membranes, assuming a membrane thickness of 1.5 nm per layer. (b) Predicted detector signal for the dark-field STIM detector with indicated acceptance angle. (c) Signal difference between the double-layer and the single-layer membrane as a function of the detector acceptance angle.

In order to understand the physics behind the STIM signal, the program Stopping and Range of Ions in Matter (SRIM) was used for Monte Carlo simulations [26]. When simulating thin membranes, it is important to use the monolayer collisions calculation type. Otherwise inaccurate results could be obtained as the collisions will be averaged over the mean free path. The simulation was run with 50,000 ions at energies of either 15 or 30 keV. The CNM was approximated as a carbon layer with a thickness between 0.3 and 13 nm. From the SRIM output file of all transmitted ions, an angular distribution was generated (see Figure 3a). The simulation results, obtained for different values of the CNM thickness show that the mean value of the scattering angle is reduced for thinner membranes. This is intuitive as the ions undergo fewer collision while traversing through the sample, leading to less accumulated deflection.

The following data treatment is intended to explain the STIM signal for CNMs of different thickness. Since the detector only converts ions that are scattered beyond the acceptance angle α, it is important to consider the cumulative sum of all ions beyond the angle α. An example of this data processing is shown in Figure 3. From a scattering distribution (Figure 3a) the ions are summed up to a theoretical STIM signal (Figure 3b). This was done for sample thicknesses of 1.5 nm (corresponding to a single-layer CNM) and 3 nm (a double-layer CNM). In the extreme case of an acceptance angle of 0 mrad (i.e., a plain metal sheet as conversion plate), all transmitted ions create a signal that can be subsequently detected. At larger acceptance angles the overall intensity decreases (Figure 3b). The signal decreases faster for a thinner membrane than for a thicker membrane towards larger angles. This makes sense, as thinner membranes lead to smaller deflection angles. Thus, the transmitted ions hit the hole, rather than creating a signal on the metal conversion plate. The difference (blue area in Figure 3b) between both signals, however, does not stay constant throughout the range of the acceptance angle. The plot in Figure 3c shows the difference in STIM signal between 3 nm thin and 1.5 nm thin CNMs for different acceptance angles. While the difference in the signal is low at small and large angles, the simulation results show that it reaches a maximum at an acceptance angle of 10 mrad.

Depending on the acceptance angle of the detector, maximum brightness or maximum contrast between different thicknesses are adjustable. The simulation results suggest that smaller acceptance angles are ideal for applications where brightness is important, such as end-point detection for, for example, nanopores. Especially on extremely thin membranes such as CNMs, milling processes are very fast and need a clearly defined signal change when the membrane is milled through. With acceptance angle and ETD settings tuned correctly, an abrupt signal change from bright to dark could easily trigger a stop command. If thickness variations, such as folds, or contaminations are of interest, larger acceptance angles serve better, at the cost of losing overall image brightness.

### Experimental evaluation of signal and contrast using the HIM

**Figure 4:**
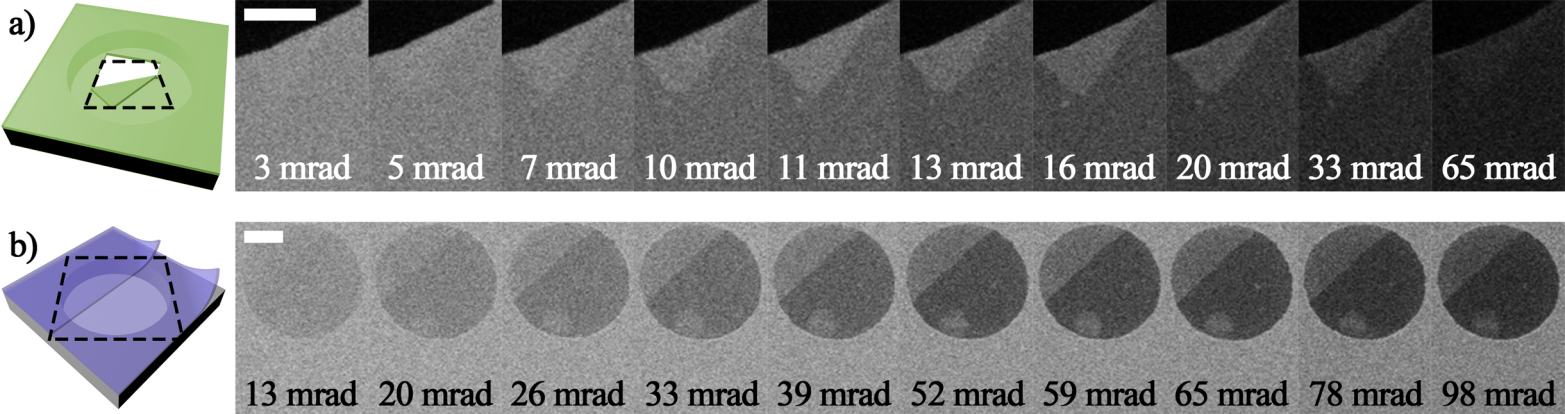
Dark-field STIM images of (a) a thin and (b) a thick membrane. The membrane in (a) shows a rupture (black area) and a folded CNM. The example in (b) shows a CNM spanning over a hole in the Quantifoil support. Both are showing the signal intensity of single- and double-layer membranes. For the thicker membrane in (b), the signal of maximum contrast between one layer and two layers is shifted towards larger acceptance angles. Scale bars are 400 nm.

Figure 4 shows a series of HIM images of CNMs using the dark-field STIM detector configured with a range of difference acceptance angles. For each image, a different conversion plate with a hole size corresponding to the acceptance angle is installed on the holder. The two sets of images were taken of membranes of different thicknesses. For each set, microscope and ETD settings were optimized and conditions were kept constant for all images within the set.

Figure 4a shows a freestanding membrane that was intentionally ruptured by a high-flux helium ion beam prior to imaging. The image shows the rupture, the membrane, and a folded layer of the CNM. At small acceptance angles, the membrane yields a high-intensity signal, while the overall intensity drops when increasing the acceptance angle. This is in agreement with the SRIM simulations. At low and at high acceptance angles, single layer and double layer are hard to distinguish. Maximum contrast, that is, maximum signal difference between the single layer and the double layer, is achieved at an acceptance angle of 11 mrad.

Figure 4b shows a thicker membrane. The images show a single layer and a double layer of the membrane spanning over a hole in the Quantifoil support. Overall intensity and contrast between the different layers are similar to those of the thin membrane, but shifted to larger acceptance angles. Single layer and double layer are hard to distinguish at an acceptance angle of 13 mrad. This angle yielded almost the maximum contrast for the thin CNM. The maximum contrast for the thick CNM is reached at an acceptance angle of about 52 mrad.

### Thickness measurements using dark-field STIM and Monte Carlo simulations

As shown in Figure 4, the maximum contrast between single- and double-layer membrane shifts towards larger angles for a thicker membrane. Quantifying this effect allows for the determination of the membrane thickness by comparing the measured grey levels to SRIM simulations as demonstrated in the following.

**Figure 5:**
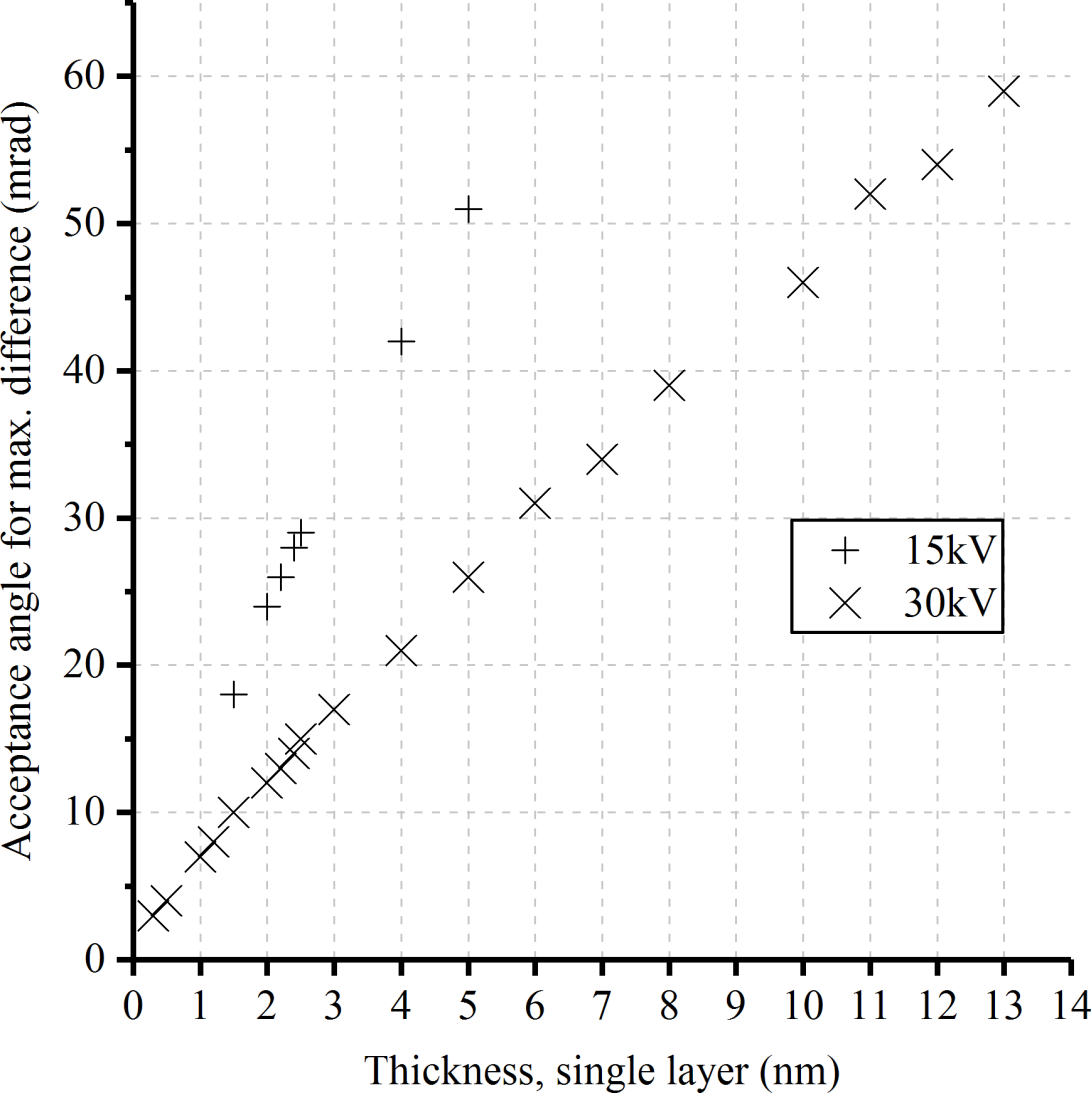
Acceptance angle at which the maximum signal difference between a double layer and a single layer is expected as a function of the layer thickness. The data was extracted from graphs such as those shown in Figure 3c.

In Figure 3c the existence of a maximum signal difference between a single- and a double-layer membrane was predicted. In this case, the thickness of the membrane was assumed to be 1.5 nm resulting in a maximum at an acceptance angle of 10 mrad. To determine the membrane thicknesses of both imaged samples in Figure 4, many curves of the signal difference, as in Figure 3c, were simulated for different membrane thicknesses. The acceptance angle of the maximum was extracted from each signal difference graph and plotted as a function of the membrane thickness. The result is shown in Figure 5. Each point in the graph is based on two simulations with the thickness corresponding to a single- and a double-layer membrane. The simulations were run using kinetic ion energies of 30 and 15 keV. This plot serves as a reference chart for thickness determination of the examined samples.

**Figure 6:**
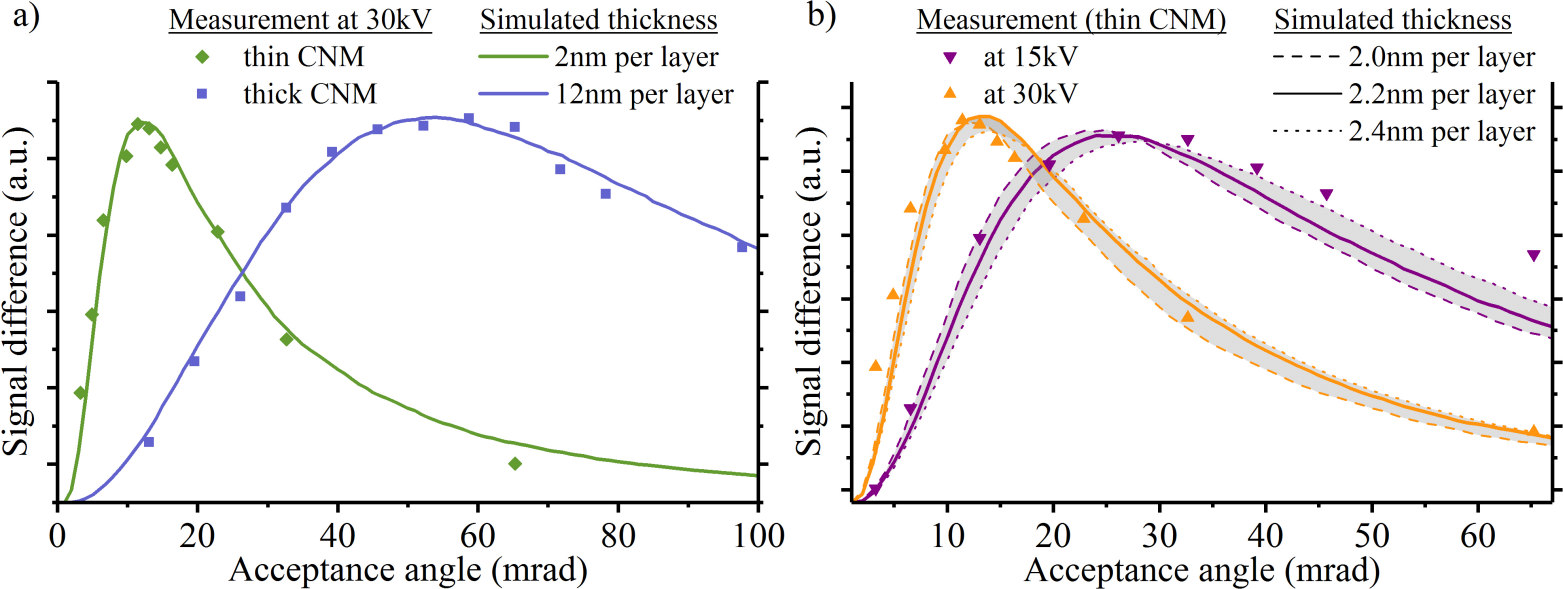
Thickness determination by dark-field STIM and Monte Carlo simulations. (a) The measured image grey values of single- and double-layer regions were extracted from both samples of Figure 4. According to the experimental data, the curve with the closest maximum was chosen based on Figure 5. Both curves are plotted for visualization. (b) Measurement of the same CNM (thin) at different energies to determine the variation of thickness results. The best-fitting curve at 15 keV yields a thickness of 2.4 nm per layer, at 30 keV it yields 2.0 nm per layer.

The grey levels from the experiments were processed analogous to the simulation data. From each image in Figure 4, mean grey values were determined for the single layer and the double layer. The difference between these signals was plotted as a function of the acceptance angle. Figure 6a shows the experimental results for both membranes. To determine the thickness, the position of the maximum needs to be compared to the simulations (see Figure 5). For visualization, the best-matching signal curve was plotted for each membrane. By this, the thickness of the thin and the thick membrane was determined to be 2 and 12 nm, respectively.

To estimate the error of the thickness determination, a CNM is imaged at two different kinetic ion energies (15 and 30 keV) and the results are compared to Monte Carlo simulations (see Figure 6b). The simulation of a 2.0 nm thick CNM (dashed lines) agrees well with the measurement at 30 keV energy for the thin membrane. This is reasonable as it is the same membrane as shown in Figure 6a. When evaluating the contrast at a nearby area at 15 keV ion energy, however, 2 nm is not the optimum thickness to reproduce the experimental contrast at 15 keV. At this lower incident ion energy, a thickness of 2.4 nm (dotted lines) matches best the measurement values. Still, the results suggest that the CNM thickness can be determined with sub-nanometer accuracy using dark-field STIM measurements. A mean CNM thickness value of 2.2 nm is a compromise between the two cases, and the error of this particular measurement is 9%.

To test the validity of the determined thickness and the approach described above, the results from the STIM dark-field imaging are compared to X-ray photoelectron spectroscopy (XPS) and energy-filtered transmission electron microscopy (EFTEM) data, both being established techniques for the thickness measurement of thin films. All three presented methods, STIM, XPS, and EFTEM, require an assumption about density and composition of the material in order to calculate absolute thicknesses, so graphite was chosen as a well-characterized material.

XPS collects the signal from a measurement spot with a diameter of a few hundred micrometers. It has, therefore, a much lower lateral resolution than STIM. It requires the membrane to be placed on a solid bulk material. Therefore, from the same membrane batch used for STIM measurements, thin and thick CNMs were transferred onto a 300 nm Au film on a mica substrate for XPS analysis. The thickness was calculated from the attenuation of the Au 4f_7/2_ photoelectrons by the membrane on top. Details about the calculation can be found in the Supporting Information of the manuscript from Turchanin et al. [27]. In order to provide comparability of the results to STIM and EFTEM, the attenuation length was assumed to be 27 Å, the same as that of graphite [28]. The XPS results yield values of 2.1 nm for the thin CNM and 12.4 nm for the thick CNM. Both values are in good agreement with 2.2 nm and 12.0 nm measured by STIM (Table 1).

**Table 1:** Thickness values determined by STIM and other established methods.

	STIM	XPS	EFTEM 60 keV	EFTEM 80 keV

thin CNM	2.2 nm	2.1 nm	1.8 nm	1.7 nm
thick CNM	12.0 nm	12.4 nm	10.5 nm	11.0 nm

**Figure 7:**
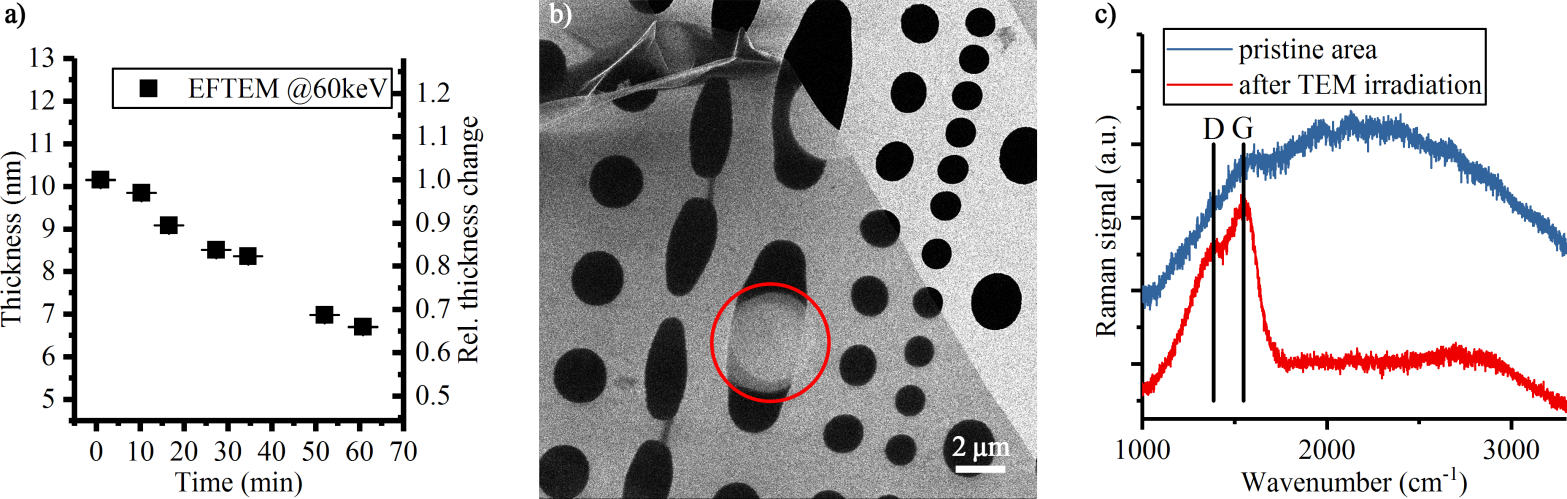
Alteration of a CNM by EFTEM at 60 keV beam energy. (a) The thickness decreases during EFTEM imaging, error bars refer to the standard error of the mean. The sample was exposed to the beam for 1 h and measured intermittently. (b) HIM SE image of an area on the CNM after taking two consecutive EFTEM images (red circle). After the area was exposed to the beam for 3 min, the area turned conductive as the bright signal in SE imaging indicates. (c) Raman measurement on the indicated area in (b) and a reference spectrum of a freestanding, non-irradiated area on the same sample. After irradiation the photoluminescence background drops and D and G peaks appear, showing a graphitization of the area exposed to EFTEM.

As a second method for comparison, EFTEM was performed on the samples after STIM measurements. EFTEM enables thickness mapping with a high lateral resolution. In EFTEM, elastically scattered electrons in a zero-loss energy-filtered image are compared to the total electron intensity of a non-filtered image [29]. It requires the estimation of an attenuation length λ, which was calculated by the Iakoubowskii model (Equation 9 in [30]), assuming here that the films exhibit the mass density of pure graphite. EFTEM measurements were done at kinetic energies of 60 and 80 keV to rule out possible deviations from the calculation of the attenuation length. Both energies are below the energy threshold for atom displacement in carbon membranes and should not lead to knock-on damage on the membrane [31]. The use of an energy filter reduces the beam energy at the sample by 3 keV, leading to electron mean free paths of 86.1 nm for 77 keV and 70.5 nm for 57 keV. The results for both thin and thick CNMs are listed in Table 1. The thicknesses are noticeably smaller than those obtained from STIM and XPS analyses. During EFTEM imaging it became clear that the membrane thickness decreased with increasing measurement time. In an extreme test scenario, the same area on the thick CNM was irradiated in TEM for 60 min while the thickness was monitored by EFTEM imaging (Figure 7a). The thickness decreases by 35% over 60 min. It should be noted, however, that one hour is very long compared to the usual measurement times of half a minute to a few minutes. Furthermore, the neglect of impurities in the CNM results in incorrect mean free paths λ and leads to a thickness deviation from the first image on.

To further investigate the observed phenomenon in the EFTEM measurement, HIM and Raman spectroscopy were performed on the samples after the EFTEM experiments. The SE image in Figure 7b shows that membrane turned brighter in the areas previously imaged with EFTEM, which is indicative of a higher electric conductivity, as demonstrated in the following. In Figure 7c, a Raman measurement of this spot is compared to a non-exposed part of the membrane. The non-exposed membrane shows barely visible D and G bands on a strong photoluminescence background as it is reported for amorphous hydrogenated carbon films [32]. After exposure to TEM, the photoluminescence drops and the D and G bands become more pronounced, similar to a film of high-temperature annealed nanographite [33]. It was previously demonstrated in TEM that, at an energy of 80 keV, a graphitization of free-standing amorphous carbon can be induced [34]. It is also known that CNMs can turn from the amorphous state into nanocrystalline graphene upon heating [25]. It was observed that this comes with a loss of atoms, which is also apparent in this experiment by the decreasing membrane thickness (see Figure 7a).

The results from both methods, XPS and EFTEM, confirm the thickness measurement by STIM. While XPS and STIM are close regarding the absolute values, EFTEM shows a deviation. The approximation of the electron mean free path and the alteration of the CNM upon irradiation with high electron flux in EFTEM can explain this deviation.

## Conclusion

This work demonstrates the capability of an easy-to-build dark-field STIM detector for ion microscopes. The dark-field STIM detector enables the imaging of very thin carbon nanomembranes with a high signal-to-noise ratio, which could not be achieved using the conventional ETD detector in the HIM. The evaluation of the contrast mechanism suggests that the detector is suitable for end-point detection, for example to drill small holes into thin foils. Thickness variations, caused by double layers or folds in membranes can be clearly visualized with the detector. In addition, thickness measurements with sub-nanometer precision are possible on thin foils or membranes when the contrast is evaluated and compared to Monte Carlo simulations. A comparison with established thickness measurements confirms the STIM results.

## Experimental

A dark-field scanning transmission ion microscopy (STIM) holder was designed for a Zeiss Orion Plus helium ion microscope (HIM). All HIM and STIM experiments were conducted on the Zeiss Orion Plus using helium ions with kinetic energies of 30 and 15 keV. A 10 µm aperture was selected and the working distance was kept identical for all images at 9.8 mm. The beam current ranged from 0.3 to 0.5 pA between different sets of images but stayed constant within a set of images. The grid of the Everhart–Thornley detector was kept at 500 V while photomultiplier gain, brightness, and image intensity were adjusted for each set of images to keep the signal on all images within the dynamic range of the detector. Images were acquired at a dwell time of 0.5 µs with line integration of 64 lines. The field of view was set to 10 µm for the thin carbon membrane and to 15 µm for the thick membrane.

The carbon nanomembranes (CNMs) were formed by low-energy electron irradiation of aromatic self-assembled monolayers (thin CNMs) or spin coated layers of aromatic molecules (thick CNMs). The electron irradiation leads to a crosslinking of the aromatic molecules and results in an amorphous membrane whose thickness is defined by the initial aromatic layer. CNMs were transferred onto TEM grids (Quantifoil Multi A) using a sacrificial layer of poly(methyl methacrylate) [35]. Thin CNMs were prepared from terphenylthiol precursor molecules, thick CNMs were obtained from CNM Technologies GmbH (Bielefeld, Germany). The CNMs used for this experiment offer a low conductivity to give a negligible SE contribution.

Monte Carlo simulations of the ion trajectories were run using the software package TRIM in the program Stopping and Range of Ions in Matter (SRIM) [26]. The “Surface Sputtering/Monolayer Collision Steps” calculation was selected due to the limited thickness of the membranes to ensure that the collisions in each monolayer were considered. 50000 ions at 15 and 30 keV energy were simulated. The membranes were assumed to consist of pure carbon with the density of graphite of 2.253 g/cm^2^. The annular deflection angles of the transmitted ions were obtained from the transmitted ions output file and their distribution was determined using a frequency count in steps of 1 mrad.

X-ray photoelectron spectroscopy was conducted on an Omicron Multiprobe instrument using a monochromatized Al Kα X-ray source (1486.7 eV, 225 W) and an Omicron Sphera hemispherical electron energy analyzer at a constant analyzer energy of 12.5 eV. The data was acquired under a photoelectron emission angle of 13° with respect to the surface normal.

Energy-filtered transmission electron microscopy was conducted at energies of 60 and 80 keV on a C_s_-corrected JEOL JEM-ARM200F equipped with a cold field emission gun. A GATAN GIF-Quantum ER image filter and a 2k × 2k CCD camera (GATAN UltraScan) were used during EFTEM operation.
